# Acute Septic Arthritis of the Knee

**Published:** 1902-07-19

**Authors:** 


### ACUTE SEPTIC ARTHRITIS OF THE KNEE.

Acting on the good old surgical maxim of making a free opening into inflamed tissue, Mr. Walter Whitehead has [1] been treating a case of septic arthritis of the knee by what he describes as the original method of laying the joint completely open. The case was one in which the internal semilunar cartilage had been removed, and although this had been done under the most rigid code of aseptic rules, yet within five days it became obvious that something had gone wrong, local pain and constitutional symptoms having developed. Free drainage and irrigation were promptly and systematically conducted, but without lessening in the least the gravity of the symptoms, and the condition of the patient became exceedingly critical—high temperature, rapid pulse, delirium, and extreme exhaustion giving evidence that unless prompt measures were at once adopted the patient would rapidly sink. The orthodox line of procedure, Mr. Whitehead says, would undoubtedly have been immediate amputation of the thigh. A transverse incision was, however, made through the skin over the centre of the patella; the patella was sawn across; the joint was then fully flexed and thoroughly opened in the same line, and the crucial ligaments were freely divided. All joint surfaces and cavities were then scraped with a spoon, and all the surfaces freely swabbed with turpentine, the whole being then carefully packed with iodoform gauze. The joint was then well flexed beyond a right angle, and retained in that position, being well packed around with wood wool. The result was immediate and most striking: all pain disappeared, the temperature fell, and the patient had refreshing sleep. The dressings were left undisturbed for two days and on their removal the tissues looked clean and granulations were visible. After this they were changed daily. It is interesting to note that on the fifth day, as the granulations appeared to be somewhat exuberant, strips of oil silk were applied over the exposed surface, and that although this had the desired effect, it caused the temperature to rise, as if the retention of the discharge under the oil silk had caused some absorption to take place. On removing the oil silk the temperature fell again. On the thirtieth day after the original operation, and the fifteenth after the adoption of the open method, the leg was straightened and fixed on a back splint, the upper fragment of the patella being at the same time removed. Finally the remaining cicatrising surface was covered in with a large Thiersch graft. Commenting on this case Mr. Whitehead says that the essential and tangible advantage of this method is that it affords the only opportunity of completely evacuating the joint of its deadly contents, for by any other method there is always a risk of a residual ferment being left behind.

[1] Brit. Med. Jour., June 21.

